# Simultaneous regulation of *F5H* in COMT‐RNAi transgenic switchgrass alters effects of *COMT* suppression on syringyl lignin biosynthesis

**DOI:** 10.1111/pbi.13019

**Published:** 2018-10-24

**Authors:** Zhenying Wu, Nengfei Wang, Hiroshi Hisano, Yingping Cao, Fengyan Wu, Wenwen Liu, Yan Bao, Zeng‐Yu Wang, Chunxiang Fu

**Affiliations:** ^1^ Shandong Provincial Key Laboratory of Energy Genetics Key Laboratory of Biofuels Qingdao Institute of Bioenergy and Bioprocess Technology Chinese Academy of Sciences Qingdao Shandong China; ^2^ Key Lab of Marine Bioactive Substances The First Institute of Oceanography State Oceanic Administration Qingdao Shandong China; ^3^ Noble Research Institute, LLC Ardmore OK USA; ^4^ Institute of Plant Science and Resources Okayama University Kurashiki Okayama Japan

**Keywords:** caffeic acid *O*‐methyltransferase, coordinated effects, ferulate 5‐hydroxylase, lignin biosynthesis, *Panicum virgatum* L., switchgrass

## Abstract

Ferulate 5‐hydroxylase (F5H) catalyses the hydroxylation of coniferyl alcohol and coniferaldehyde for the biosynthesis of syringyl (S) lignin in angiosperms. However, the coordinated effects of F5H with caffeic acid *O*‐methyltransferase (COMT) on the metabolic flux towards S units are largely unknown. We concomitantly regulated *F5H* expression in *COMT*‐down‐regulated transgenic switchgrass (*Panicum virgatum* L.) lines and studied the coordination of F5H and COMT in lignin biosynthesis. Down‐regulation of *F5H* in COMT‐RNAi transgenic switchgrass plants further impeded S lignin biosynthesis and, consequently, increased guaiacyl (G) units and reduced 5‐OH G units. Conversely, overexpression of *F5H* in COMT‐RNAi transgenic plants reduced G units and increased 5‐OH units, whereas the deficiency of S lignin biosynthesis was partially compensated or fully restored, depending on the extent of *COMT* down‐regulation in switchgrass. Moreover, simultaneous regulation of *F5H* and *COMT* expression had different effects on cell wall digestibility of switchgrass without biomass loss. Our results indicate that up‐regulation and down‐regulation of *F5H* expression, respectively, have antagonistic and synergistic effects on the reduction in S lignin resulting from COMT suppression. The coordinated effects between lignin genes should be taken into account in future studies aimed at cell wall bioengineering.

## Introduction

Lignin, which mainly deposits in secondary cell wall of vascular plants, exists as a complicated phenolic heteropolymer cross‐linking with cell wall polysaccharides to form a complex matrix. Lignin is required for structural support, water transport and plant defence in plant growth and development (Boerjan *et al*., 2003). *p*‐Coumaryl, coniferyl and sinapyl alcohols are the major precursors of lignin synthesized through the hydroxylation and methylation of derivatives from the phenylpropanoid pathway. The lignin units derived from the three monolignols are known as *p*‐hydroxyphenyl (H), guaiacyl (G) and syringyl (S) units (Boerjan *et al*., 2003). Their proportions vary with plant species and tissue types (Chapple *et al*., 1992).

**Figure 1:**
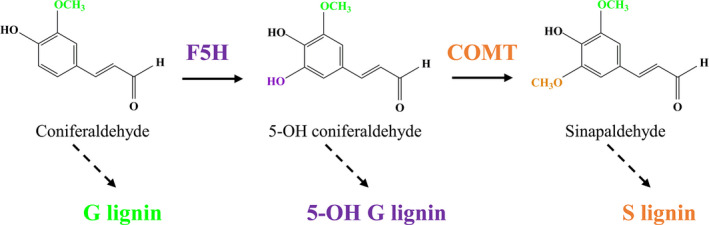
Schematic of locations of ferulate 5‐hydroxylase (F5H) and caffeic acid *O*‐methyltransferase (COMT) in the lignin biosynthetic pathway.

Over recent decades, a combinatorial approach of forward and reverse genetics has been used to investigate the lignin biosynthetic pathway widely (Bonawitz and Chapple, 2010; Fu *et al*., 2011a; Humphreys and Chapple, 2002; Lewis and Yamamoto, 1990; Vanholme *et al*., 2013). According to the currently accepted model of lignin biosynthesis, caffeyl CoA *O*‐methyltransferase is responsible for methylating the 3‐hydroxyl group of lignin intermediates leading to G lignin production, whereas caffeic acid *O*‐methyltransferase (COMT) is involved in 5‐*O*‐methylation leading to S lignin production. Previous studies have suggested that COMT mainly participates in the conversion of 5‐OH coniferaldehyde/5‐OH coniferyl alcohol to sinapaldehyde/sinapyl alcohol. Disruption of *COMT* can result in a severe reduction in S units accompanying with consequent incorporation of unusual 5‐OH G units in numerous plant species (Goujon *et al*., 2003; Palmer *et al*., 2008; Ralph *et al*., 2001; Rastogi and Dwivedi, 2008; Vignols *et al*., 1995). The accumulation of S units, however, can be affected by other lignin biosynthetic enzymes. For example ferulate 5‐hydroxylase (F5H, CYP84A1) is a cytochrome P450‐dependent monooxygenase that hydroxylates coniferaldehyde and coniferyl alcohol into 5‐OH coniferaldehyde and 5‐OH coniferyl alcohol for the subsequent formation of S lignins (Figure 1). Therefore F5H coupled with COMT can divert coniferaldehyde and coniferyl alcohol towards S lignin precursors and thereby alter both G‐ and S‐units deposition (Humphreys *et al*., 1999). The knockout of *F5H* in *Arabidopsis* produces a *fah1* mutant comprising almost entirely of G unit and barely any S units, whereas overexpression of *F5H* results in low G and high S units (Chapple *et al*., 1992; Meyer *et al*., 1998). As expected, up‐regulation or down‐regulation of *F5H* in poplar, tobacco and alfalfa can lead to lignins consisting of significantly altered S/G ratios (Franke *et al*., 2000; Reddy *et al*., 2005). In contrast, the function of F5H has yet to be investigated in monocots.

Lignin engineering is currently focusing on incorporation of atypical lignin monomers in plant cell walls for improving the digestibility of lignocellulosic biomass (Lee *et al*., 2017). It has been indicated that down‐regulation of *F5H* in *Arabidopsis cinnamyl alcohol dehydrogenase* (*CAD*) double mutant (*cadc* and *cadd*) can produce lignins derived exclusively from polymerization of coniferaldehyde (Anderson *et al*., 2015). The alteration of lignin composition of a *fah1 cadc cadd* triple mutant, however, has no negative effects on plant growth and development, whereas the lignin polymers enrich in coniferaldehyde units and the cell wall digestibility is substantially increase. In contrast, overexpression of *F5H* in the CAD‐deficient *Arabidopsis* mutant causes plant dwarfism, and the cell walls of *C4H:F5H cadc cadd* plants enrich sinapaldehyde units (Anderson *et al*., 2015). In addition, overexpression of *F5H* in COMT‐deficient *Arabidopsis* mutant, *omt1*, can result in lignin polymers with dramatically reduced amounts of G and S units as well as substantially increased 5‐OH G units (Vanholme *et al*., 2010; Weng *et al*., 2010). Unfortunately, overexpression of *F5H* in this mutant affects plant development severely, which is consistent with the previous observation in *C4H:F5H cadc cadd Arabidopsis* plants. Unlike the *omt1* null mutant, *COMT* transcripts are not entirely suppressed in COMT‐antisense or ‐RNAi transgenic plants. It remains unclear if a large percentage of changes in G and S units without biomass loss can be achieved in *COMT*‐down‐regulated transgenic plants via concomitant regulation of *F5H* expression. Overall, these results suggest that simultaneous regulation of lignin biosynthetic genes can lead to lignin polymers with diverse composition and, therefore change cell wall digestibility and plant growth.

Here, we characterized the function of *F5H* in switchgrass (*Panicum virgatum* L.), a dual‐purpose forage and biofuel crop, and found that *F5H* was a crucial factor that affected both G and S lignin biosynthesis. Simultaneous down‐regulation of *F5H* and *COMT* synergistically reduced S lignin biosynthesis in switchgrass, whereas overexpression of *F5H* in the severely COMT‐suppressing background partially compensated for the loss of S lignin. Furthermore, overexpression of *F5H* in the moderately COMT‐suppressing background was able to fully restored S lignin biosynthesis of switchgrass. Moreover, the transgenic switchgrass lines with diverse lignin composition and elevated saccharification efficiency of cell walls may be valuable for different purposes of cell wall bioengineering in the future.

## Results

### Identification and isolation of switchgrass *F5H* sequences

**Figure 2:**
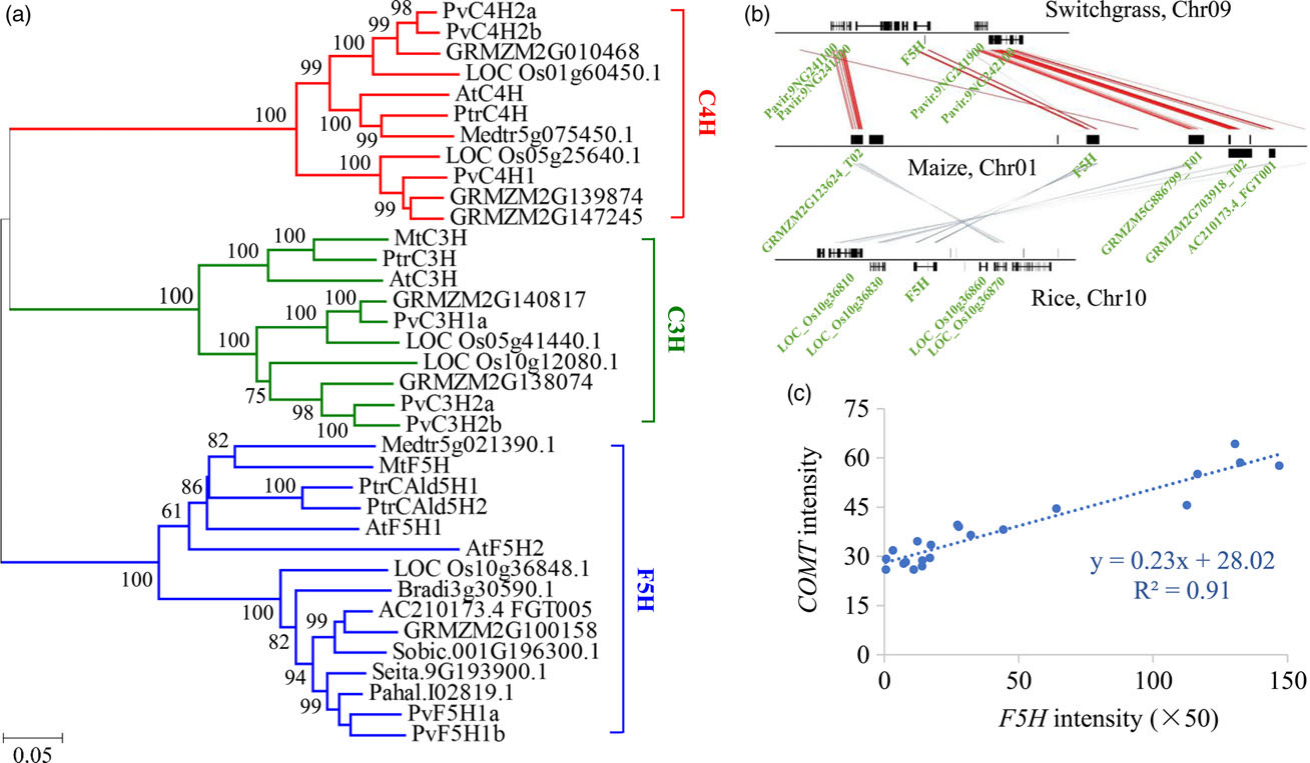
Molecular characterization of *PvF5H*. (a) Phylogenetic analysis of the three P450s (C4H, C3H and F5H) in the lignin biosynthetic pathway. A maximum likelihood tree was constructed in PhyML version 3.0 on the basis of multiple alignments of the deduced protein sequences from switchgrass, maize, sorghum, rice, *Brachypodium distachyon*,* Arabidopsis thaliana*,* Medicago truncatula* and *Populus trichocarpa*. Sequence data from this article can be found in Phytozome and/or Genbank under the following accession numbers: switchgrass Pavir.9NG241700.1 (PvF5H1a), Pavir.9KG138400.1 (PvF5H1b), Pavir.Fb01856.1 (PvC4H1), Pavir.5KG602000.1 (PvC4H2a), Pavir.5NG607400.1 (PvC4H2b), Pavir.3KG265800.1 (PvC3H1a), Pavir.5KG602000.1 (PvC3H2a), Pavir.5NG607400.1 (PvC3H2b); maize AC210173.4_FGT005 (F5H), GRMZM2G100158 (F5H), GRMZM2G139874 (C4H), GRMZM2G147245 (C4H), GRMZM2G010468 (C4H), GRMZM2G140817 (C3H), GRMZM2G138074 (C3H); sorghum Sobic.001G196300.1 (F5H); rice LOC_Os10g36848.1 (F5H), LOC_Os05g25640.1 (C4H), LOC_Os01g60450.1 (C4H), LOC_Os05g41440.1 (C3H), LOC_Os10g12080.1 (C3H); *B. distachyon* Bradi3g30590.1 (F5H); *A. thaliana* At4g36220 (AtF5H1), At5g04330 (AtF5H2), AT2G30490 (AtC4H), AT2G40890 (AtC3H); *M. truncatula* Medtr8g076290.1 (MtF5H), ABC59086.1 (MtC3H); and *P. trichocarpa* Potri.005G117500.1 (PtrCald5H1), Potri.007G016400.1(PtrCald5H2), Potri.013G157900.1 (PtrC4H), Potri.006G033300.1 (PtC3H). (b) Collinear relationships of *F5H* orthologs in genomes of switchgrass, maize and rice. A chromosomal region of *PvF5H1a* including 40‐kb flanking sequences were aligned with the corresponding orthologous sequences in maize (100 kb) and rice (40 kb). (c) Correlations between expression levels of *F5H* and *COMT* in different tissues and organs of switchgrass. The representative probesets of *F5H* (AP13ITG56842_at) and *COMT* (KanlowCTG00989_s_at) were retrieved from the switchgrass gene expression atlas. COMT, caffeic acid *O*‐methyltransferase.

To gain insight into the *F5H* functions in switchgrass, we first identified *F5H* sequences from switchgrass. The assembled switchgrass genome (*P. virgatum* v4.1, Phytozome) contains a pair of *F5H* genes (*PvF5H1a* and *1b*) located on chromosome 2 and share over 95% sequence identities between genes (Figure S1). The sequences of the three cytochrome P450 monooxygenases in the lignin biosynthetic pathway, F5H, cinnamate 4‐hydroxylase (C4H) and *p*‐coumaroyl shikimate 3′‐hydroxylase (C3H), were downloaded from eight genome‐sequenced species (switchgrass, maize, sorghum, rice, *Brachypodium distachyon*,* Arabidopsis thaliana*,* Medicago truncatula* and *Populus trichocarpa*) for the analysis of phylogenetic relationships. The phylogenetic tree showed that PvF5H1a and 1b clustered together in a group containing the typical functional F5Hs (Figure 2a). Moreover, the collinearity analysis of *F5H* orthologs in genome of switchgrass, maize and rice also revealed a close relationship in gene evolution and functions as well (Figure 2b). Therefore, we isolated the full‐length cDNA sequences of *PvF5H1a* from switchgrass for further functional investigation. Sequence alignment showed that the open reading frame of *PvF5H1a* shared 99% sequence identity with a previously isolated switchgrass *F5H* (NCBI accession No. AB608019) (Figure S1). Publicly available switchgrass gene expression atlas data revealed that *PvF5H* had relative high signal intensity in well‐lignified tissues and organs (Figure S2). Moreover, a high positive correlation was found between the expression pattern of *PvF5H* and *PvCOMT* (Figure 2c).

### Effects of *F5H* regulation on lignin biosynthesis

**Figure 3:**
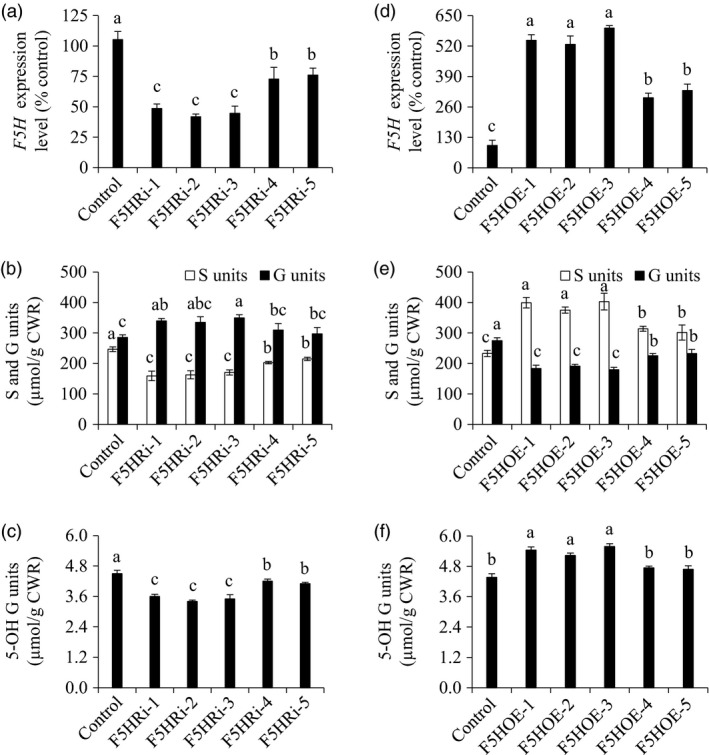
Characterization of F5HRi and F5HOE transgenic switchgrass plants. (a) Quantitative real‐time PCR analysis of *F5H* transcript abundances in the F5HRi transgenic lines. (b) S and G lignin monomer yield in the F5HRi transgenic lines. (c) 5‐OH G lignin monomer yield in the F5HRi transgenic lines. (d) Quantitative real‐time PCR analysis of *F5H* transcript abundance in the F5HOE transgenic lines. (e) S and G lignin monomer yield in the F5HOE transgenic lines. (f) 5‐OH G lignin monomer yield in the F5HOE transgenic lines. The control plants for TF5HRi and TF5HOE transgenic lines were generated with pANIC8D and pANIC6D empty vectors respectively. Stems at the R1 stage were collected. Switchgrass *UBQ* was used as the reference for normalization. CWR, cell wall residue. Values are mean ± SE (*n* = 3). Means with the different letter are significantly different (One‐way ANOVA, Duncan's test, *P *<* *0.05).

To examine the effects of *F5H* down‐regulation on the biosynthesis of G and S units in switchgrass, we first produced *F5H*‐suppressing transgenic plants with the single genotypic embryogenic callus line to exclude the potential influence of the genetic background of switchgrass on lignin biosynthesis (Figure 3a). The 485‐bp fragment of the *PvF5H1a* gene that can target both *PvF5H1a* and *PvF5H1b* was employed to make a hairpin structure for post‐translational gene silencing. The control plants were produced with the pANIC8D empty vector which was used as the backbone for constructing F5H‐RNAi vector. Lignin composition analysis revealed a 12.9%–35.4% reduction in S units as well as a 4.2%–22.5% increase in G units in F5HRi transgenic lines compared with those of the control plants (Figure 3b). Moreover, 5‐OH G units were also reduced in the above transgenic lines (Figure 3c).

To examine the effects of *F5H* up‐regulation on the biosynthesis of G and S units in switchgrass, we produced F5H*‐*overexpressing transgenic plants (Figure 3d). The control plants were produced with pANIC6D empty vector which was used as the backbone for constructing the F5H‐OE vector. Our results revealed a 17.4%–22.5% increase in S units as well as a 15.3%–34.7% reduction in G units in F5HOE transgenic switchgrass lines compared with those of the control plants (Figure 3e). As a consequence of *F5H* up‐regulation, 5‐OH G units increased in the above transgenic lines (Figure 3f).

### Effects of *COMT* down‐regulation on *F5H* expression levels

The methylation of phenylpropanoid meta‐hydroxyl at the 5‐position is catalysed by COMT which is a well‐characterized key enzyme for the biosynthesis of S lignin in angiosperms. Two COMT isoforms, *PvCOMT1* and 2, are found on chromosomes 2 and 6 of switchgrass respectively. Sequence alignment revealed that the PvCOMT1 protein previously isolated from switchgrass (Fu *et al*., 2011b) shares 84% identity with PvCOMT2. Gene Atlas analysis showed that *PvCOMT1* had approximately 200‐fold higher signal intensity in different tissues than *PvCOMT2* (Figure S3). Therefore, the 558‐bp fragment of the *PvCOMT1* gene was employed to make a hairpin structure. To evaluate whether autonomous crosstalk occurs between *COMT* and *F5H* in the COMT‐suppressing background, we produced the COMT‐RNAi transgenic switchgrass lines to examine the expression levels of both *COMT* and *F5H*. The control plants were produced with the pANIC8B empty vector. A total of seven independent transgenic lines were subjected to analysis of *F5H* expression levels. Variation in *F5H* transcript abundances were observed among these *COMT* down‐regulated transgenic switchgrass lines (Figure S4).

### Simultaneous down‐regulation of *F5H* and *COMT* synergistically reduced S lignin biosynthesis

**Figure 4:**
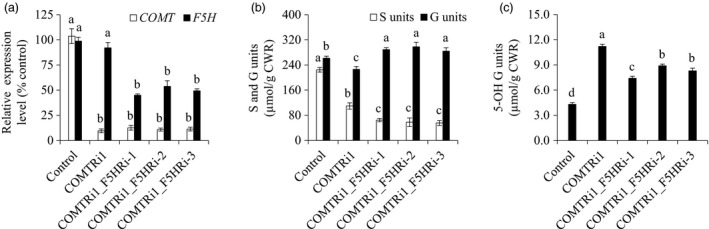
Characterization of transgenic switchgrass plants with *F5H* down‐regulation in the caffeic acid *O*‐methyltransferase (COMT)‐suppressing background. (a) Quantitative real‐time PCR analysis of *COMT* and *F5H* transcript abundances in the COMTRi1‐F5HRi transgenic lines. (b) S and G lignin monomer yield in the COMTRi1‐F5HRi transgenic lines. (c) 5‐OH G lignin monomer yield in the COMTRi1‐F5HRi transgenic lines. The control plants were generated with the pANIC8D empty vector. Stems at the R1 stage were collected. Switchgrass *UBQ* was used as the reference for normalization. CWR, cell wall residue. Values are mean ± SE (*n* = 3). Means with the different letter are significantly different (One‐way ANOVA, Duncan's test, *P *<* *0.05).

Given the location of F5H in the network of the lignin biosynthetic pathway (Figure 1), we suspected that *F5H*‐down‐regulation may have a synergistic effect on S lignin reduction in the COMT‐suppressing background. To test our hypothesis, we re‐transformed the F5H‐RNAi cassette into a COMTRi1 transgenic line in which *COMT* expression was severely suppressed, and generated the double‐transformed switchgrass plants containing both COMT‐RNAi and F5H‐RNAi cassettes. Six independent double transformants were generated, and two of the six transgenic lines showed varying *COMT* expression levels compared with that of COMTRi1 (Table S1). Three double‐transformed switchgrass lines with comparable *COMT* expression level to that of COMTRi1 plants were chosen for further biochemical analysis (Figure 4a). Lignin composition analysis revealed that the transgenic lines with the double*‐*down‐regulation of *F5H* and *COMT* exhibited a lower proportion of S units than the COMTRi1 transgenic plants (Figure 4b). Compared with the single down‐regulation of *COMT* in COMTRi1 and control plants, the double‐down‐regulated transgenic lines showed a significant increase in G units (Figure 4b). Furthermore, down‐regulation of *F5H* in the COMT‐suppressing background reduced the accumulation of 5‐OH G units that resulted from the COMT down‐regulation; however, these double‐down‐regulated transgenic lines still showed the higher proportion of 5‐OH G units than the control plants (Figure 4c).

### Overexpression of *F5H* compensated for the loss of S lignin resulted from *COMT* suppression

**Figure 5:**
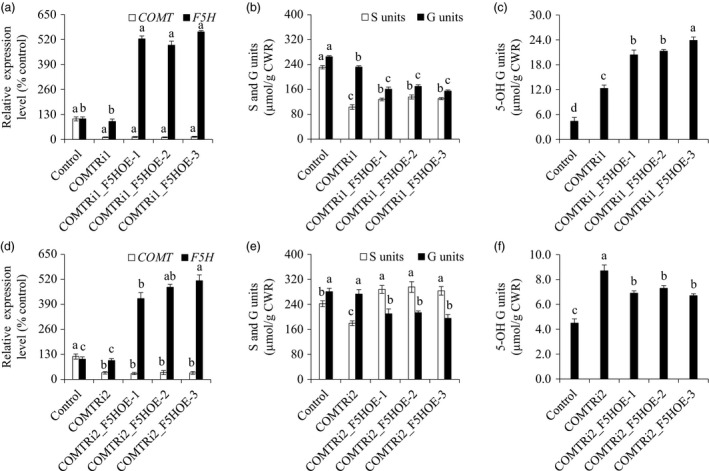
Characterization of transgenic switchgrass plants with *F5H* overexpression in the caffeic acid *O*‐methyltransferase (COMT)‐suppressing background. (a) Quantitative real‐time PCR analysis of *COMT* and *F5H* transcript abundances in the COMTRi1‐F5HOE transgenic lines. (b) S and G lignin monomer yield in the COMTRi1‐F5HOE transgenic lines. (c) 5‐OH G lignin monomer yield in the COMTRi1‐F5HOE transgenic lines. (d) Quantitative real‐time PCR analysis of *COMT* and *F5H* transcript abundance in the COMTRi2‐F5HOE transgenic lines. (e) S and G lignin monomer yield in the COMTRi2‐F5HOE transgenic lines. (f) 5‐OH G lignin monomer yield in the COMTRi2‐F5HOE transgenic lines. The control plants were generated with the pANIC6D empty vector. COMTRi1: the COMT‐RNAi line with severe down‐regulation of COMT; COMTRi2: the COMT‐RNAi line with moderate down‐regulation of COMT. Stems at the R1 stage were collected. Switchgrass *UBQ* was used as the reference for normalization. CWR, cell wall residue. Values are mean ± SE (*n* = 3). Means with the different letter are significantly different (One‐way ANOVA, Duncan's test, *P *<* *0.05).

To study whether up‐regulation of *F5H* in the COMT‐suppressing background can also affect S lignin accumulation, we first re‐transformed F5H‐OE cassette into the COMTRi1 transgenic line. Seven independent double transformants were generated, and two of the seven transgenic lines showed varying *COMT* expression levels compared with that of COMTRi1 (Table S1). As described previously, only the double‐transformed lines with comparable *COMT* expression level to the COMTRi1 plants were chosen for further biochemical analysis (Figure 5a). Lignin composition analysis showed that the transgenic lines with up‐regulated *F5H* and down‐regulated *COMT* expressions contained a lower proportion of G units and a higher proportion of 5‐OH G units than those of the COMTRi1 transgenic plants (Figure 5b,c). Compared with the single down‐regulated of *COMT* in COMTRi1 plants, the lower amount of S units resulting from *COMT* suppression was partially compensated in the double‐transformed lines (Figure 5b).

Given that COMTRi1 was the line with severe down‐regulation of *COMT*, we next employed a moderately down‐regulated *COMT* transgenic line, COMTRi2, to further study the antagonistic effects of *F5H* overexpression on S lignin biosynthesis in the *COMT*‐suppressing background (Figure 5d). Five independent double transformants were generated, and one of the five transgenic lines showed varying *COMT* expression levels compared with that of COMTRi2 (Table S1). Overexpression of *F5H* in the COMTRi2 transgenic switchgrass line resulted in a significant reduction in G units as expected (Figure 5e). Most strikingly, the reduction in S units resulting from *COMT* suppression was fully restored in the double‐transformed transgenic lines (Figure 5e). As a consequence, a relatively low proportion of 5‐OH G units were observed in the COMTRi2_F5HOE transgenic lines compared with that of the COMTRi2 plants; however, their 5‐OH G units levels were still higher than those of the control and F5HOE transgenic plants (Figures 3f and 5f). To further confirm that the negative effect of moderate down‐regulation of *COMT* on S lignin biosynthesis was still well sustained in the double‐transformed lines, we conducted a soluble phenolics profiling analysis by LC‐MS/MS and identified 5‐OH coniferyl alcohol glycoside, a derivative from the substrates of COMT, present in the COMTRi2_F5HOE transgenic lines (Table S2; Figure S5). Moreover, this compound specifically accumulated in the COMT‐suppressing background including COMTRi1, COMTRi2, COMTRi1_F5HOE and COMTRi2_F5HOE transgenic switchgrass plants, but not in F5HRi, F5HOE and empty vector control plants (Figure S5).

Taken together, these results imply that increasing the concentration of substrates for COMT by overexpressing *F5H* in the COMT‐suppressing background may compensate for the lack of COMT enzyme due to down‐regulation of *COMT*. To test this hypothesis, we measured COMT activity against 5‐OH coniferyl alcohol in crude plant extracts of the control, COMTRi1 and COMTRi2 plants. Our results showed that the turn‐over efficiency of crude COMT enzyme extracts prepared from COMT‐RNAi transgenic switchgrass plants was dramatically elevated by increasing the concentration of substrate (Figure S6).

### Effects of diverse lignin composition on plant growth and cell wall digestibility

**Figure 6:**
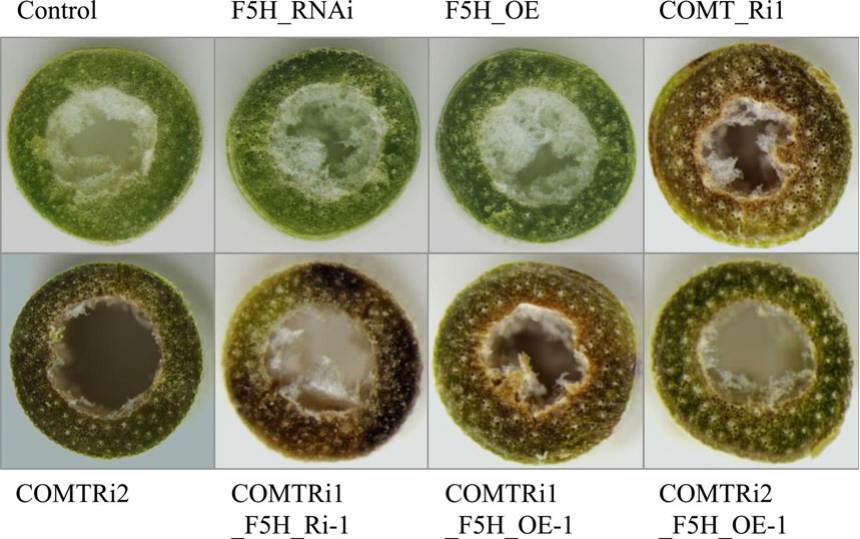
Cross sections of internodes from transgenic switchgrass plants. The control plants were generated with the pANIC empty vector. Stems at the R1 stage were collected and the different internodes were separated.

To evaluate whether the coordination between F5H and COMT affects switchgrass growth and development, we characterized the phenotype of the F5HRi, F5HOE, COMTRi and their double‐transformed plants. Alteration of *F5H* expression either in the wild type background or in the COMT‐suppressing background had no effects on plant growth and development (Figure S7). However, the double‐transformed switchgrass plants exhibited brown‐coloured stems (Figure 6). Furthermore, coloration analysis of the cross sections of internodes showed that the brown‐coloured pigment was the typical characterization of COMT suppression but not F5H*‐*alteration (Figure 6). No difference between the control and transgenic switchgrass plants was detected in the above ground biomass (Table 1).

**Table 1:** Effects of modification of *COMT* and *F5H* on biomass, cell wall digestibility and lignin content of transgenic switchgrass plants

	Dry matter biomass (g/plant)	Enzymatic hydrolysis efficiency (%)	Acetyl bromide lignin (mg/g CWR)
Control	21.7 ± 1.5^a^	32.2 ± 0.9^e^	234.6 ± 3.4^c^
COMTRi1	20.5 ± 3.9^a^	40.7 ± 1.1^c^	217.5 ± 1.6^b^
COMTRi2	23.5 ± 1.5^a^	36.4 ± 0.4^d^	230.1 ± 2.4^c^
F5HRi‐1	20.9 ± 2.2^a^	33.4 ± 0.7^ed^	224.8 ± 4.9^c^
F5HOE‐1	22.4 ± 2.1^a^	32.5 ± 1.0^ed^	240.5 ± 3.6^c^
COMTRi1_F5HRi‐1	21.3 ± 4.3^a^	51.1 ± 0.9^a^	195.8 ± 5.3^a^
COMTRi1_F5HRi‐2	20.3 ± 3.1^a^	50.5 ± 0.8^a^	188.8 ± 4.4^a^
COMTRi1_F5HRi‐3	18.8 ± 2.1^a^	51.2 ± 1.4^a^	187.2 ± 1.5^a^
COMTRi1_F5HOE‐1	23.1 ± 3.3^a^	44.1 ± 1.3^bc^	219.6 ± 1.9^b^
COMTRi1_F5HOE‐2	17.3 ± 3.6^a^	45.4 ± 0.5^b^	208.1 ± 0.7^ab^
COMTRi1_F5HOE‐3	19.5 ± 2.0^a^	43.0 ± 1.9^bc^	215.4 ± 1.9^b^
COMTRi2_F5HOE‐1	20.6 ± 2.9^a^	30.8 ± 0.9^e^	232.6 ± 4.3^c^
COMTRi2_F5HOE‐2	21.9 ± 1.6^a^	31.9 ± 2.9^e^	232.7 ± 1.0^c^
COMTRi2_F5HOE‐3	24.2 ± 3.8^a^	32.1 ± 0.8^e^	225.4 ± 3.7^c^

The transgenic and control plants were harvested after six months of growth in the greenhouse. Values are mean ± SE (*n* = 3). Means with the same letter are not significantly different (One‐way ANOVA, Duncan's test, *P *<* *0.05).

Given that lignin is a crucial factor negatively affecting anthropogenic utilization of lignocellulosic biomass, we next evaluated the effect of altered lignin biosynthesis on cell wall digestibility. As anticipated, cell wall saccharification efficiencies of the COMTRi1 and COMTRi2 transgenic switchgrass plants were 26.4% and 13.0% greater than that of the control plants. Neither up‐regulation nor down‐regulation of *F5H* affected saccharification efficiency significantly (Table 1). Altering *F5H* expression levels in the COMTRi1 transgenic line, however, resulted in up to 11.6%–25.8% increases in saccharification efficiency compared with that of the COMTRi1 transgenic plants (Table 1). In contrast, the saccharification efficiencies of COMTRi2_F5HOE transgenic switchgrass lines were lower than those of COMTRi2 transgenic plants, but resembled those of the control plants (Table 1). In addition, the influence of acetyl bromide (AcBr) lignin content on digestibility of cell walls was investigated, revealing a strong negative correlation (*r* ^2^ = 0.884) between lignin content and saccharification efficiency (Figure S8). No significant correlation was observed between saccharification efficiency and 5‐OH G lignin level and S/G ratio (Figure S8).

## Discussion

Genetic regulation of either *F5H* or *COMT* can affect S lignin biosynthesis severely and thereby improve forage digestibility, bioethanol production and woody pulping efficiency (Baxter *et al*., 2014; Chen and Dixon, 2007; Chen *et al*., 2004; Jung *et al*., 2012; Pilate *et al*., 2002; Shen *et al*., 2012). An effective method of lignin modification by RNAi‐mediated down‐regulation of COMT in switchgrass was successful in reducing S lignin biosynthesis and improving the conversion efficiency of lignocellulosic biomass into bioethanol (Fu *et al*., 2011a). On the other hand, the function of F5H remains largely elusive in monocot species. In this study, we characterized the function of F5H in switchgrass and revealed the coordination of F5H and COMT in the biosynthesis of S lignin.

Ferulate 5‐hydroxylase hydroxylates coniferaldehyde and coniferyl alcohol to 5‐OH coniferaldehyde and 5‐OH coniferyl alcohol, which is the master step in S lignin biosynthesis. Disruption or down‐regulation of *F5H* in *Arabidopsis*, alfalfa and rice can reduce S units and enrich G units in lignin polymers (Meyer *et al*., 1998; Reddy *et al*., 2005; Takeda *et al*., 2017). Conversely, overexpression of *F5H* in *Arabidopsis*, tobacco, poplar and rice can enrich S units and reduce G units (Franke *et al*., 2000; Meyer *et al*., 1998; Takeda *et al*., 2017). Switchgrass *PvF5H1* has high amino acid sequence identity to the recently characterized rice *OsCAld5H*, and the collinearity analysis revealed *PvF5H1* as the ortholog of *OsCAld5H,* implying that *PvF5H1* could function in the S lignin biosynthesis as well. In line with the previous studies, both S and G lignin levels were significantly altered in transgenic switchgrass plants with either up‐regulation or down‐regulation of *PvF5H1*, indicating that F5H plays a crucial role in lignin biosynthesis in switchgrass. Compared with *F5H*, disruption of *COMT* results in a substantial loss of S lignin units in the *Arabidopsis omt1* mutant, whereas overexpression of *COMT* has no effects on S lignin biosynthesis, suggesting that F5H, rather than COMT, appears to be a rate‐limiting step. Notably, a relative low level of down‐regulation for *F5H* in F5H‐RNAi transgenic switchgrass lines was sufficient to reduce S lignin biosynthesis significantly. In contrast, a similar loss in S units was not achieved until the transcript abundances of *COMT* were reduced by more than 80% in COMT‐RNAi transgenic switchgrass lines (Fu *et al*., 2011a; Figures 3, 4, 5). These results imply that the expression levels of *F5H* in switchgrass might be regulated more strictly during cell wall lignification. In addition, a recent study has shown that the phosphorylation of COMT in poplar can switch off its activity, demonstrating a novel regulation mechanism in S lignin biosynthesis (Wang *et al*., 2015). Unfortunately, little information on post‐translational modifications of F5H and COMT is available in switchgrass. Therefore, it would be worthwhile to investigate post‐translational modifications of F5H and COMT to refine our understanding of the complex regulatory mechanisms in S lignin biosynthesis.

Monolignols are synthesized through a complex metabolic network in plants. Therefore, it is difficult to predict the impacts of simultaneous manipulation of more than one enzyme in lignin biosynthetic pathway (Pincon *et al*., 2001; Zhao and Dixon, 2011). However, our results indicate that down‐regulation of *F5H* and *COMT* had a synergistic effect on S lignin biosynthesis in switchgrass. Moreover, the pattern of G lignin accumulation was similar to that of F5HRi transgenic switchgrass plants, but 5‐OH G lignin accumulation resembled that of COMTRi1 transgenics. In addition, previous studies have shown that overexpression of *F5H* in *Arabidopsis omt1* mutant can increase 5‐OH G units incorporation in lignin polymers and reduce both G and S lignin accumulation dramatically (Vanholme *et al*., 2010; Weng *et al*., 2010). Similar results were observed in a highly down‐regulated *COMT* transgenic switchgrass line with concomitant overexpression of *F5H*. Given the fact that *COMT* transcripts are not entirely eliminated in the COMT‐RNAi transgenic switchgrass lines, the potential dosage effect of *COMT* expression has to be considered in these double‐transformed plants. Strikingly, overexpression of *F5H* in a background with moderate down‐regulated of *COMT* was able to fully restore S lignin biosynthesis. Moreover, the level of 5‐OH G units in double‐transformed switchgrass plants was still higher than that of control plants, but lower than that of the corresponding COMTRi2‐single transgenic plants. Based on the above results, we speculate that overexpression of *F5H* in the COMT‐down‐regulated background can significantly elevate 5‐OH coniferaldehyde/5‐OH coniferyl alcohol influx and thereby compensate for the decrease in turn‐over efficiency of COMT due to the reduction in COMT expression. The data of a COMT *in vitro* enzyme activity assay further supports our hypothesis. In addition, we screened numerous COMT‐RNAi transgenic switchgrass lines and found that *COMT* down‐regulation had the potential to trigger a significant increase in *F5H* expression when the biosynthesis of S lignins was disrupted in some transgenic switchgrass lines (Figure S4). Thus the antagonistic effects of up‐regulation of *F5H* in the COMT‐down‐regulated background on S lignin biosynthesis have to be considered carefully in the practice of lignin bioengineering.

A significant increase of 5‐OH G units and cell wall digestibility has been achieved by overexpression of *F5H* in the COMT‐deficient Arabidopsis mutant (Weng *et al*., 2010). Our results showed that up‐regulating *F5H* expression in the *COMT* severely down‐regulated transgenic switchgrass significantly increased saccharification efficiency of cell walls compared with that of the empty vector control. Conversely, up‐regulating *F5H* expression in the *COMT*‐moderately down‐regulated transgenic switchgrass did not improve saccharification efficiency of cell walls. Furthermore, no correlation was observed between cell wall saccharification efficiency and 5‐OH G lignin level and S/G ratio; however, the saccharification efficiency was negatively correlated with AcBr lignin content. Therefore, our results imply that other factors besides lignin composition may still have important influences on cell wall digestibility. In addition, downregulating *F5H* expression in the severely *COMT*‐suppressing background of switchgrass remarkably elevated saccharification efficiency of cell walls compared with that of the single down‐regulation of *COMT*. These results suggest that the concomitant alteration of F5H and COMT expression levels in switchgrass plants indeed has potential to improve feedstock utilization of bioenergy crops in the future.

## Experimental procedures

### Plant materials and growth conditions

We used Alamo, a lowland type switchgrass cultivar, for lignin modification. According to the criteria described by Hardin *et al*. (2013), the development of our switchgrass plants were divided into five elongation stages (E1, E2, E3, E4 and E5) and three reproductive stages (R1, R2 and R3). Plants were grown in a greenhouse with 16 h light (390 μE/m^2^/S).

### Identification and cloning of *PvF5H*

*PvF5H1a* and *1b* were identified by blasting previously published switchgrass *F5H* sequences (GeneBank accession no: AB608019) against the switchgrass genome database v4.1 (http://www.phytozome.org/). MEGA 5 software suite (http://www.megasoftware.net/) was employed to conduct alignment of multiple sequences and phylogenetic tree analysis of F5H orthologs downloaded from switchgrass, maize, sorghum, rice, *B. distachyon*,* A. thaliana*,* M. truncatula* and *P. trichocarpa*. A maximum likelihood tree was constructed in PhyML version 3.0 (http://atgc.lirmm.fr/phyml/) on the basis of multiple alignments of deduced F5H protein sequences. Core‐orthologous gene pairs in switchgrass, maize and rice were employed to define orthologous blocks as described by Bai *et al*. (2016). The chromosomal region of *PvF5H1a* including 40 kb flanking sequences were aligned with the corresponding orthologous sequences in maize (100 kb) and rice (40 kb). Gene collinearity analysis of *PvF5H1a* was performed as described by Bai *et al*. (2016). The expression patterns of *PvF5H* and *COMT* were retrieved from the switchgrass Gene Expression Atlas (http://switchgrassgenomics.noble.org/). *PvF5H* was isolated from switchgrass stem tissues by reverse transcription polymerase chain reaction (RT‐PCR) based on the sequence of *F5H1a* downloaded from phytozome v12 and was subjected to sequencing and further studies.

### Generation of transgenic switchgrass plants

The primers used for the cloning of fragments of COMT‐RNAi, F5H‐RNAi and F5H‐OE were designed based on the code sequence of the isolated *PvCOMT* and *PvF5H* (Table S3). The final binary vectors of pANIC8B‐COMTRi, pANIC8D‐F5HRi and pANIC6D‐F5HOE were constructed by LR recombination reactions (Invitrogen, Shanghai, China), and transferred into *Agrobacterium tumefaciens* strain *AGL1* using the freezing/heat‐shock method.

A high‐quality, single genotype embryogenic callus line induced from an Alamo seed was employed for *Agrobacterium*‐mediated transformation following the procedure described by Wu *et al*. (2016). Transgenic switchgrass lines were grown in the greenhouse at 26 °C with 16 h light (390 μE/m^2^/S). In addition, the calli induced from inflorescences of the selected COMT‐RNAi transgenic lines were used for the re‐transformation of F5H‐RNAi and F5H‐OE constructs into the COMT‐suppressing background. Hygromycin and bialaphos were used as the selectable reagents to generate COMTRi‐F5HRi and COMTRi‐F5HOE transgenic switchgrass plants.

### Expression levels of *F5H* and *COMT* in transgenic switchgrass plants

The positive transgenic lines were identified by genomic PCR with specific *hph* and *bar* primers. The fragments of 375 and 242 bp were the expected sizes of PCR products for *hph* and *bar* respectively (Table S3). Stems at the R1 stage were collected from each plant and ground in liquid nitrogen. Total RNA extracted by Tri‐Reagent (Invitrogen) from approximate 0.2 g stem samples were subjected to reverse transcription with Superscript III Kit (Invitrogen) after treatment with TURBO™ DNase I (Ambion, Austin, TX). The remaining samples were lyophilized for soluble phenolics, lignin and cell wall digestibility analyses. The expression levels of *F5H* and *COMT* were analysed by quantitative real‐time PCR (qRT‐PCR) as described by Wu *et al*. (2016). The primers used for qRT‐PCR were listed in Table S3. The cycle thresholds were determined using an ABI PRISM 7900 HT sequence detection system (Applied Biosystems, Foster City, CA), and the data were normalized using the level of switchgrass *Ubq2* transcripts (GenBank accession no: HM209468).

### Determination of lignin content and composition

Soluble extracts were removed from the ground lyophilized stem samples by three successive extractions with chloroform/methanol (2 : 1 v/v), methanol and water at room temperature as described by Chen and Dixon (2007), and the remaining cell wall residues (CWRs) were lyophilized. The extractive‐free CWRs were then used to quantify lignin content and composition. The AcBr method was employed to quantify lignin content (Hatfield *et al*., 1999). The thioacidolysis method was used to determine lignin composition (Lapierre *et al*., 1995).

### Identification and quantification of soluble phenolics

Soluble phenolics were extracted twice from 30.0 ± 0.05 mg of the lyophilized above‐ground stem samples with 1.0 mL 50% methanol plus 1.5% acetic acid for 3 h at room temperature each time (Fu *et al*., 2011b). Identification and quantification of 5‐OH coniferyl alcohol glycoside was performed by LC‐PDA/ESI‐MS/MS according to a previously described method (Fu *et al*., 2011b). All the mass spectra were acquired using a Bruker Esquire LC equipped with an electrospray ionization (ESI) source. Mass spectra from positive‐ and negative‐ion ESI were recorded over the range of 50–2200 *m*/*z*. To confirm the aglycone structure of the deduced compound, the vacuum‐dried methanolic extracts of switchgrass internodes were resolved with 3 mL of 5 mg/mL β‐glucosidase in citric acid buffer (pH = 5.5) and the reaction was incubated at 37 °C overnight (Tian and Dixon, 2006). The β‐glucosidase hydrolysis products were vacuum‐dried, re‐dissolved in 1.0 mL 80% methanol and were identified by comparing their retention time, and UV‐visible and mass spectra with the corresponding standard compounds. The reference standard of 5‐OH coniferyl alcohol was synthesized by the Chemistry Research Solution LLC (PA, USA).

### Enzyme activity assay

The stems of wild type switchgrass plants collected at the R1 stage were homogenized in liquid nitrogen. Powdered tissue (about 500 mg) was extracted for 3 h at 4 °C in extraction buffer (Fu *et al*., 2011a). The samples were centrifuged at 17 900 ***g*** for 20 min at 4 °C, and the extracts were desalted on PD‐10 columns (Pharmacia, Shanghai, China). Activities of COMT against 5‐OH coniferyl alcohol in crude plant extracts were determined as described by Liu *et al*. (2012).

### Biomass measurement and internode coloration observation

The positive transgenic switchgrass plants were subjected to morphological analysis. The controls were generated from a population including the plants derived from the transgenic plants with pANIC8B, pANIC8D and pANIC6D empty vectors. Transgenic and control plants were harvested after 6‐months of growth in a greenhouse and dried in an oven at 40 °C for 1 month to evaluate the above‐ground dry matter biomass yield.

The second internode collected from the stem at the R1 stage were free‐hand sectioned with a razor blade and pictures of unstained samples were immediately taken under an Olympus *SZX*12‐Fluorescent Stereo Microscope system (Olympus, Tokyo, Japan) for internode coloration characterization.

### Determination of cell wall digestibility

The switchgrass stems (R1 stage) collected in a paper bag were oven‐dried for 7 days at 40 °C. The dried stem samples were ground through a Thomas model 4 Wiley^®^ mill with a 1‐mm sieve and used for CWR preparation as described by Chen and Dixon (2007). The protocol (LAP‐009, Enzymatic Saccharification of Lignocellulosic Biomass; http://www.nrel.gov/biomass/analytical_procedures.html) described by National Renewable Energy Laboratory were then used to determine saccharification efficiency of the extractive‐free CWRs. The phenol‐sulphuric acid assay was employed to measure the amount of fermentable sugars (Dubois *et al*., 1956). The ratio of sugars released by enzymatic hydrolysis to the amount of total sugars present in cell wall materials before the enzymatic hydrolysis treatment was determined as saccharification efficiency of cell walls.

### Statistical analysis

Primary transgenic switchgrass plants were propagated by transferring the same number of tillers into each pot. Three copies of each line were grown in 1‐gallon pots. Stems at the R1 stage were collected from the three copies of each transgenic line. Two technical replicates were conducted for lignin and cell wall digestibility analyses of each sample. The mean values were used for statistical analysis. Data from each trait were subjected to one‐way analysis of variance (ANOVA). The significance of treatments was tested at the *P *=* *0.05 and 0.01 level. Standard error of the mean is provided in all figures and tables as appropriate. Means with the different letter are significantly different (One‐way ANOVA, Duncan's test, *P *<* *0.05).

## Conflict of interest

The authors declare no conflict of interest.

## Supporting information


**Figure S1** Alignment of switchgrass *F5H* nucleic acid sequences.
**Figure S2** Gene expression atlas analysis of *PvF5H*.
**Figure S3** Gene expression atlas analysis of *PvCOMT1* and *PvCOMT2*.
**Figure S4** Quantitative RT‐PCR analysis of *PvF5H* transcript abundances in COMT‐RNAi transgenic switchgrass plants.
**Figure S5** 5‐OH coniferyl alcohol glucoside yield in methanolic extracts of stems of control and transgenic switchgrass plants.
**Figure S6** Extractable COMT enzyme activity in stems of control and COMT‐RNAi transgenic switchgrass plants.
**Figure S7** Morphological characterization of transgenic switchgrass plants.
**Figure S8** Relationships between saccharification efficiency and lignin content and composition.
**Table S1** Varying *COMT* and *F5H* expression levels in the double transgenic switchgrass lines.
**Table S2** Identification of 5‐OH coniferyl alcohol glycoside by LC‐MS/MS in COMT‐RNAi transgenic switchgrass plants.
**Table S3** Primers used in this study.
