# Supplementary material for: Simultaneous regulation of F5H in COMT‐RNAi transgenic switchgrass alters effects of COMT suppression on syringyl lignin biosynthesis
Source: Plant Biotechnol J. 2018 Oct 24;17(4):836–45. doi: 10.1111/pbi.13019 (PMC6419721; doi:10.1111/pbi.13019)
Supplement: Supplementary file 1 — Figure S1 Alignment of switchgrass F5H nucleic acid sequences. Figure S2 Gene expression atlas analysis of PvF5H. Figure S3 Gene expression atlas analysis of PvCOMT1 and PvCOMT2. Figure S4 Quantitative RT‐PCR analysis of PvF5H transcript abundances in COMT‐RNAi transgenic switchgrass plants. Figure S5 5‐OH coniferyl alcohol glucoside yield in methanolic extracts of stems of control and transgenic switchgrass plants. Figure S6 Extractable COMT enzyme activity in stems of control and COMT‐RNAi transgenic switchgrass plants. Figure S7 Morphological characterization of transgenic switchgrass plants. Figure S8 Relationships between saccharification efficiency and lignin content and composition. Table S1 Varying COMT and F5H expression levels in the double transgenic switchgrass lines. Table S2 Identification of 5‐OH coniferyl alcohol glycoside by LC‐MS/MS in COMT‐RNAi transgenic switchgrass plants. Table S3 Primers used in this study. [file PBI-17-836-s001.doc]

**Supporting information**

Simultaneous regulation of F5H in COMT-RNAi transgenic switchgrass alters effects of COMT suppression on syringyl lignin biosynthesis

Zhenying Wu, Nengfei Wang, Hiroshi Hisano, Yingping Cao, Fengyan Wu, Wenwen Liu, Yan Bao, Zeng-Yu Wang and Chunxiang Fu

The following supporting information is available for this article:

**Figure S1** Alignment of switchgrass *F5H* nucleic acid sequences.

**Figure S2** Gene expression atlas analysis of *PvF5H*.

**Figure S3** Gene expression atlas analysis of *PvCOMT1* and *PvCOMT2*.

**Figure S4** Quantitative RT-PCR analysis of *PvF5H* transcript abundances in COMT-RNAi transgenic switchgrass plants.

**Figure S5** 5-OH coniferyl alcohol glucoside yield in methanolic extracts of stems of control and transgenic switchgrass plants.

**Figure S6** Extractable COMT enzyme activity in stems of control and COMT-RNAi transgenic switchgrass plants.

**Figure S7** Morphological characterization of transgenic switchgrass plants.

**Figure S8** Relationships between saccharification efficiency and lignin content and composition.

**Table S1** Varying *COMT* and *F5H* expression levels in the double transgenic switchgrass lines.

**Table S2** Identification of 5-OH coniferyl alcohol glycoside by LC-MS/MS in COMT-RNAi transgenic switchgrass plants.

**Table S3** Primers used in this study.

**Figure S1** Alignment of switchgrass *F5H* nucleic acid sequences.

**Figure S2** Gene expression atlas analysis of *PvF5H*.

**Figure S3** Gene expression atlas analysis of *PvCOMT1* and *PvCOMT2*.

**Figure S4** Quantitative RT-PCR analysis of *PvF5H* transcript abundances in COMT-RNAi transgenic switchgrass plants. The control plants were generated with pANIC8B empty vector. Stems at the R1 stage were collected. Switchgrass *UBQ* was used as the reference for normalization. Value are mean ± SE (n=3).

**Figure S5** 5-OH coniferyl alcohol glucoside yield in methanolic extracts of stems of control and transgenic switchgrass plants. Stems at the R1 stage were collected. Value are mean ± SE (n=3).

**Figure S6** Extractable COMT enzyme activity in stems of control and COMT-RNAi transgenic switchgrass plants. COMTRi1: the COMT-RNAi line with severe downregulation of COMT; COMTRi2: the COMT-RNAi line with moderate downregulation of COMT. Stems at the R1 stage were collected. Value are mean ± SE (n=3).

**Figure S7** Morphological characterization of transgenic switchgrass plants. Representative plants from each group are shown. The control plants were generated with pANIC empty vector.

**Figure S8** Relationships between saccharification efficiency and lignin content and composition.

Data from Figure 3-5 and Table 1 were used for correlation analysis. (a) Correlation of saccharification efficiency and AcBr lignin content; (b) Correlation of saccharification efficiency and 5-OH G lignin level; (c) Correlation of saccharification efficiency and S/G ratios. Spearman correlation coefficients were determined between saccharification efficiency and lignin content and composition.

**Table S1** Varying *COMT* and *F5H* expression levels in the double transgenic switchgrass lines.

|  | Relative *COMT* expression levels  (% control) | Relative *F5H* expression levels  （% control） |
| --- | --- | --- |
| COMTRi1 | 10.4±1.2^e^ | 92.9±5.8^e^ |
| COMTRi1_F5HRi-1 | 13.1±2.2^e^ | 44.9±1.2^f^ |
| COMTRi1_F5HRi-2 | 10.1±1.5^e^ | 53.7±3.8^f^ |
| COMTRi1_F5HRi-3 | 11.4±1.9^e^ | 49.3±2.1^f^ |
| COMTRi1_F5HRi-4 | 12.3±1.8^e^ | 51.1±3.4^f^ |
| COMTRi1_F5HRi-5 | 49.5±4.6^b^ | 55.7±2.8^f^ |
| COMTRi1_F5HRi-6 | 55.6±3.4^b^ | 46.4±3.0^f^ |
| COMTRi1_F5HOE-1 | 12.6±2.1^e^ | 523.1±15.5^ab^ |
| COMTRi1_F5HOE-2 | 10.8±1.9^e^ | 490.6±20.9^b^ |
| COMTRi1_F5HOE-3 | 13.8±2.7^e^ | 559.4±6.8^a^ |
| COMTRi1_F5HOE-4 | 13.5±1.5^e^ | 300.0±19.8^d^ |
| COMTRi1_F5HOE-5 | 10.3±0.5^e^ | 330.9±27.1^d^ |
| COMTRi1_F5HOE-6 | 44.7±5.7^bc^ | 426.5±30.7^c^ |
| COMTRi1_F5HOE-7 | 84.6±4.4^a^ | 332.7±22.6^d^ |
| COMTRi2 | 34.7±6.6^cd^ | 98.5±9.5^e^ |
| COMTRi2_F5HOE-1 | 30.6±5.6^d^ | 420.3±30.2^c^ |
| COMTRi2_F5HOE-2 | 35.7±10.3^cd^ | 478.7±15.2^b^ |
| COMTRi2_F5HOE-3 | 33.9±7.9^cd^ | 512.9±30.7^ab^ |
| COMTRi2_F5HOE-4 | 32.9±3.4^cd^ | 283.5±11.9^d^ |
| COMTRi2_F5HOE-5 | 73.1±3.3^a^ | 386.6±18.1^c^ |

The transgenic and control plants were harvested after 6-month growth in the greenhouse. Value are mean ± SE (n=3). Means with the same letter are not significantly different (One-way ANOVA, Duncan’s test, *p*<0.05).

**Table S2** Identification of 5-OH coniferyl alcohol glycoside by LC-MS/MS in COMT-RNAi transgenic switchgrass plants.

| Deduced compound | UV λmax  (nm) | Positive MS/MS | | Negative MS/MS | |
| --- | --- | --- | --- | --- | --- |
|  |  | Parent ions (*m/z*) | Daughter ions (*m/z*) | Parent ions (*m/z*) | Daughter ions (*m/z*) |
| 5-OH coniferyl alcohol glycoside | 265 | 381 [M+Na]^+^  397 [M+K]^+^ | 219 [M+Na-162]^+^ | 357 [M-H]^-^ | 195 [M-162]^-^  180 [195-CH_3_]^-^ |

**Table S3** Primers used in this study.

| **Primer name** | **Forward primer** | **Revere primer** | **Functional identification** |
| --- | --- | --- | --- |
| *COMT1ORF* | CACCATGGGCTCGACCGCCGCCGACG | CTACTTGGTGAACTCGATGGCCCA | For recombinant protein assay |
| *COMT_RNAi* | CACCAGGTCCTCATGGAGAGCTGGTA | TGATCATGTCGACGTGGAAGAC | For constructing COMT-RNAi vector |
| *F5HRi* | CACCCCGCCACCATCGCCGTCTCGTA | CAGCGTCGGCGTCGTCGGGTCTTC | For constructing F5H-RNAi vector |
| *F5HOE* | CACCATGGTGGCCGTGCCCAAGGTC | TAGCTCAGTGCAAGGGGCAGTTGAG | For constructing F5H-overexpression vector |
| *Hph3/4* | AAGGAATCGGTCAATACACTACATGG | AAGACCAATGCGGAGCATATACG | For identifying positive transgenic switchgrass lines |
| *Bar* | AGTCGACCGTGTACGTCTCC | GAAGTCCAGCTGCCAGAAAC | For identifying positive transgenic switchgrass lines |
| *COMT1-3’UTR* | CGGCAAGGAGCGCTACGAGAGG | GCAGGCCAGCAGCAGGATGAAGAT | For qRT-PCR analysis of *COMT1* expression |
| *COMT2-3’UTR* | GCACAACCCCGGCGGCAAGGAG | GGCAACAAAATGACGAAGAC | For qRT-PCR analysis of *COMT2* expression |
| *F5HORF* | GCCTCTACGCGCTGGAGCTC | TCAGTACAAGGGGCAGTTGAG | For qRT-PCR analysis of *F5H* expression |
| *Ubiquitin* | TTCGTGGTGGCCAGTAAG | AGAGACCAGAAGACCCAGGTACAG | For qRT-PCR as the internal standard |
